# Caregiver assessment of patients with advanced cancer: concordance with patients, effect of burden and positivity

**DOI:** 10.1186/1477-7525-6-42

**Published:** 2008-06-02

**Authors:** Irene J Higginson, Wei Gao

**Affiliations:** 1 King's College London, School of Medicine at Guy's, King's College and St Thomas' Hospitals, Department of Palliative Care, Policy and Rehabilitation, Weston Education Centre, Cutcombe Road, London, SE5 9RJ, UK

## Abstract

**Background:**

Clinicians and researchers often have to rely on information from caregivers to assess patients with advanced cancer. This study aims to assess the validity (using patients' assessment as the gold standard) of caregiver reports of patient concerns and the roles of caregiver burden and positivity.

**Methods:**

A total of 64 advanced cancer patient and informal caregiver dyads were recruited from regional palliative care services and interviewed. Patients' outcomes were assessed with both the patient and the caregiver version of the Palliative Outcome Scale (POS); caregiver burden and positivity were collected with the Zarit Burden interview (ZBI) and three questions on achievements and relationships. The agreement between patient- and caregiver-rated POS was measured with weighted kappa statistics. The roles of caregiver burden and positivity in POS agreement were studied with logistic regression controlling for potential confounders; adjusted odds ratios were estimated from the models.

**Results:**

Agreement was substantial for pain, moderate for four items, fair for three items and slight for two items. Compared with patients self-ratings, caregivers described more problems with information given and sharing feelings and were less likely to assess the patient felt their life was worthwhile or felt good about themselves. Disagreement for three POS item ratings was significantly associated with higher caregiver burden: "feeling anxious"(OR: 4.5; 95%CI: 1.3 to 15.6), "life worthwhile"(OR: 12.4; 95%CI: 2.9 to 54.3) and "felt good" (OR: 7.7; 95%CI: 2.0 to 29.6). Caregivers with higher positivity scores were more likely to agree patients' rating of "felt good"(OR: 0.3; 95%CI: 0.1–0.9) but at increased risk of disagreeing about patient's "practical problems"(OR: 4.2; 95%CI: 1.1 to 16.6).

**Conclusion:**

Caregiver burden and positivity affect their assessments, especially of psychological patient domains and whether patients assess their life as worthwhile. Awareness of this might help clinicians and researchers better interpret caregiver assessments.

## Background

In advanced cancer the assessment of pain, symptoms and domains relevant to quality of life are an essential component of quality care. In clinical practice, the oncologist assesses the symptoms and factors affecting quality of life in order to plan treatment. However, as the cancer progresses it becomes more difficult for some patients to directly provide assessments, because they are too weak, or develop delirium [1]. Equally, in research, missing data from patients who have become too ill to record assessments or who have died become a serious problem[2,3].

In the absence of patient information, clinicians and researchers often rely on the reports of caregivers, family members and sometimes staff. Contemporary assessments from lay caregivers (and family members) are often reported as being closer to patient assessments than those of staff. However, there is variance in the literature about the extent to which caregivers agree with patients' self-ratings [4-6]. Although many studies have assessed agreement for symptoms and quality of life, few have examined agreement regarding factors increasingly relevant at the end of life, including psychological and spiritual concerns [5-7]. Better understanding of what influences agreement or disagreement between patients with advanced disease and their caregivers would be useful, for clinicians and researchers working among people with advanced cancer. It would be especially important if family members become decision makers, as is often the case in the terminal stages of cancer [8].

Caregiver burden refers to people's emotional response to the changes and demands of giving support to another. Studies among elderly patients and those with dementia have suggested that caregivers with higher levels of burden tend to be less in agreement with patients' own ratings of quality of life[6]. However, the burden of caregiving in dementia and in nursing homes is different from that in advanced cancer, where caregiving is intensive, but over a shorter period than for dementia. Further, caregiver burden is a concept that emphasizes the 'negative' components of caregiving, rather than the positive elements, which may be an important motivation for the caregiver[9]. There is increasing emphasis on resilience[5,9-11] – of both patients and caregivers – and scales assessing the positive aspects of caregiving have now begun to be developed. It may be that just as greater caregiver burden may reduce agreement, positivity of caregiving may increase agreement. It is also possible that burden and agreement are not strongly inversely correlated – as some individuals may be highly burdened but also perceive positive aspects of caregiving[12].

Understanding the roles of caregiver burden and positivity in the agreement of patient and caregiver reported ratings could help us to selectively use proxy assessment from caregivers and also effectively design intervention programs[6]. Therefore, we undertook a study: 1) to compare the patient self-ratings with lay caregiver ratings using a widely used palliative outcome scale (POS); 2) to determine whether the agreement was influenced by the level of subjective burden and positivity of caregivers.

## Methods

### Design

cross-sectional ratings recorded by patients and, separately, by their nearest lay caregiver or family member.

### Setting

Six regional palliative care services in the south of England (London and Chichester). Inclusion criteria were: advanced cancer and receiving palliative care services, either day care, home care, hospice, or hospital support teams. The patients were part of a larger study assessing the effectiveness of different models of palliative care service[13,14]. Consenting patients identified their nearest caregiver who, where possible, was also recruited, consented and interviewed. Multi-centre ethics committee approval was granted by the National Research Ethics Service, South East Research Ethics Committee.

### Data Collection

data was collected between 2000 and 2002 using trained interviewers, usually meeting consenting patients and caregivers, in their own homes, who were interviewed separately. Clinical and demographic data of patients and caregivers were collected. Patients' general health status was measured by using an item from Euro QoL-5D on a visual analogue scale (0–100)[15]. In addition, patients and caregivers separately completed the Palliative Outcome Scale (POS)[14,16]. POS was developed from extensive review of the literature and testing with both users (patients and caregivers from a range of cultures) and clinicians[16]. Independent validation of POS found it can usefully reflect practice in both inpatient palliative care settings and nursing homes[17,18]. The scale does not function to record one single underlying construct "quality of life" but it reflects the main components enshrined by Dame Cicely Saunders in the concept of "total pain" including pain and other symptoms, emotional, social, spiritual/existential and communication/information components[14]. The effect of each item on the daily life of the individual over the last three days is scored on a 5-point Likert scale ranging from 0 (not at all) to 4 (overwhelmingly). For example, "over the last three days have you been feeling anxious or worried about your illness or treatment?" The patient version of POS directly asked the patient about their symptoms and information needs whereas the caregiver version asked the caregiver to assess their view of the patients' circumstances. Details of scales are shown in the appendix.

In addition, caregivers self-reported demographic information and completed the Zarit Burden Interview (ZBI), a 22 item, 5-point Likert scale (never = 0, nearly always = 4) used widely to assess caregiver burden[19,20]. The total burden was obtained by adding the scores for all items with a range of 0 to 88, higher scores indicating greater burden. Positivity was evaluated using three 5-point Likert scale questions about the positivity of caregiving ("Do you feel a sense of achievement caring for your relative"; "Do you feel that you have developed a closer relationship with your relative since you have been caring for him/her?"; "Has caring for your relative altered your ideas about what is important in life?"). These are based on the stress-appraisal-coping model of Lazarus et al[21], the Lawton scale[22] and questions used in a survey of almost 1000 caregivers in Australia[11]. Combined they give a total score ranging from 0 to 12 with a higher score indicating a more positive view.

### Analysis

Mean and median score of patient- and caregiver-rated POS were calculated for each individual item. Due to the non-normal distribution of data, the Wilcoxon signed-rank test was used to test for differences between patients and caregivers. Internal consistency of the scale was explored using Cronbach's alpha, although we hypothesised that values for POS would not be high, because POS does not measure one underlying construct. The agreement between patient and caregiver dyads was assessed using Cicchetti-Allison type weighted Kappa [23] in conjunction with the percentage agreement (the percentage of cases for which patient and caregiver agree), as Kappa may be low even when there are high levels of agreement if there is prevalence bias (i.e. many scores are one value)[24]. Kappa tests for agreement controlling for chance agreement and its values were considered as follows: slight (0.0–0.2), fair (0.21–0.40), moderate (0.41–0.60), substantial (0.61–0.80) and perfect agreement (>0.80)[25].

The associations between POS agreement and informal caregiver burden and positivity were evaluated using multiple logistic regression. The dependent variable was an indicator of agreement for which 0 and 1 respectively denoted 'agree' (where caregiver and patient ratings were identical) and 'disagree'. Five variables (patient: cancer site; caregiver: age, gender, relationship to patient, living status) which had statistical significance (by bivariate tests) and/or clinical/biological relevance were included in the models to control for their potential effects[6,26]. Scores of caregiver burden and positivity were dichotomized as 0 and 1 (with median score as the cutoff). We chose to dichotomize the scores using median because: 1) dichotomized scores provided the best fit compared to the other types of scores (e.g. original, trichotomized); 2) median (comparing to lower and upper quartile) dichotomized scores provided the best power[27,28]. We tested the relationship between caregiver burden and positivity using Spearman correlation. But we could not be sure that in reality there was no colinearity between burden and positivity because of the conflicting findings from prior studies[9,29,30] and the limitations of our sample size. So caregiver burden and positivity were assessed separately using multivariate models regardless of the results of bivaraite analysis. Adjusted odds ratios (OR) and their 95% confidence interval (95%CI) of the higher vs. lower caregiver burden or positivity were then estimated from the models to quantify the strength of associations. Unadjusted ORs were also reported for comparison purposes. We conducted two sensitivity analyses. First we varied the cut-off for agreement so that differences less than or equal to 1 score denoted 'agree' and disagreement beyond this denoted 'disagree'. Second, we also varied the dichotomization point of caregiver burden scores and the caregiver positivity scores to the first and then the third quartiles, i.e. selecting only the least or most burdened and the least or most positive caregivers. All tests were two-sided and a P-value < 0.05 was considered statistically significant. Analyses were conducted using SAS version 9.1.3 (SAS Institute, Cary, NC).

## Results

A total of 64 patient and caregiver dyads were recruited and interviewed. Around two-thirds of the patients were male and two-thirds of the caregivers were female. Median age of patients and caregivers was between 74 and 72 years, with caregivers being slightly younger. In this group nearly all caregivers were spouses or partners and nearly all caregivers lived with the patient. Only 10 of the caregivers were working outside the home (see table 1). Table 1 also lists the descriptive statistics of total ZBI and positivity scores in the study population. The scales of burden and positivity showed high internal consistency (Cronbach's alpha: 0.85 for ZBI and 0.82 for positivity).

**Table 1:** Demographic and clinical characteristics statistics for advanced cancer patients and their caregivers

Variable	Patient n (%)*	Caregiver n (%)*
**Total**	**64**	**64**
		
**Gender**		
Male	45 (70.3)	18 (28.1)
Female	19 (29.7)	46 (71.9)
		
**Median age (min, max)**	73.8 (50, 88)	67.9 (50, 85)
**Mean age (sd, 95% CI)**	71.6 (9.1, 69.3–73.8)	69.7 (7.4, 67.9 -71.6)
		
**Relationship of caregiver to patient**		
Spouse/Partner	-	58 (90.6)
Parent	-	1 (1.6)
Child	-	3 (4.7)
Others	-	2 (3.1)
		
**Who does the patient live with?**		
Lives with spouse	57 (89.1)	-
Lives alone	1 (1.6)	-
Lives with family	5 (7.8)	-
Not known	1 (1.6)	-
		
**Employment**		
Not working outside the home	52 (81.3)	44 (68.8)
Unable to work/Stop working to care	8 (12.5)	1 (1.6)
Working outside the home	3 (4.7)	10 (15.6)
Other	1 (1.6)	9(14.1)
		
**Ethnicity**		
White	64 (100.0)	59 (92.2)
Other	0 (0.0)	5 (7.8)
		
**Primary cancer site**		
Lung	11 (17.2)	-
Gynea/Breast	7 (10.9)	-
GU/Prostate	14 (21.9)	-
Gastrointestinal	16 (25.0)	-
Other	16 (25.0)	-
		
**General health status **(Euro QoL 5D, 0–100 (best))		
Mean (SD)	61.5 (17.4)	
Median(min, max)	60 (12, 99)	
		
**Zarit total burden score **(0–88 (worst))		
Mean (SD)	-	18.5 (11.0)
Median (min, max)	-	17 (0, 60)
Cronbach's alpha	-	0.85
		
**Positivity total score **(0–12 (best))		
Mean (SD)	-	6.4 (4.0)
Median (min, max)	-	6.0(0, 12)
Cronbach's alpha	-	0.82

*Unless indicated

Table 2 presents the item-specific mean, median scales of the patient self-rated POS and the caregiver-rated POS, and the respective Cronbach alphas for patient- and caregiver-rated POS. Two phenomena emerged. First, the internal consistency, as tested by Cronbach's alpha, for patient's and caregiver's POS are both modest, suggesting that POS is a scale which does not reflect a single underlying construct but a number of constructs, as in the original development. Second, mean and median scores are very similar between patients and caregivers for six out of 10 items – pain, other symptoms, patient anxiety, family anxiety, wasted time, and practical problems. For four items the caregivers ratings described more problems than did patients: information given, sharing feeling, whether life felt worthwhile, and whether the patient felt good about themselves as a person. Caregivers reported a slightly higher (i.e. worse) and statistically significant (S = 413, p < 0.001) median total POS score than patients.

**Table 2:** Mean and median score for patients (n = 64) and caregivers (n = 64), weighted kappa and percentage agreement between patients and caregivers by POS items

POS item	Patients		Caregivers		Agreement	
	mean(SD)	Median (min, max)	mean (SD)	Median (min, max)	Weighted Kappa (95%CI)	Percentage agreement(%)

Pain	1.4(1.1)	1(0,4)	1.3(1.0)	1(0,4)	0.69(0.54–0.83)	53.1
Other symptoms	1.2(1.1)	1(0,4)	1.2(1.1)	1(0,4)	0.51(0.31–0.71)	54.7
Feeling anxious	1.0(1.2)	0(0,4)	1.0(1.0)	1(0,4)	0.44(0.20–0.67)	50.0
Family anxious	1.8(1.3)	2(0,4)	1.6(1.2)	1(0,4)	0.49(0.30–0.68)	37.5
Information given*	0.2(0.8)	0(0,4)	0.9(1.6)	0(0,4)	0.04(-0.10–0.17)	71.9
Share feeling*	0.6(1.2)	0(0,4)	1.0(1.3)	1(0,4)	0.48(0.26–0.71)	51.6
Life worthwhile**	0.5(0.8)	0(0,4)	0.9(1.0)	1(0,4)	0.31(0.03–0.58)	48.4
Felt good**	1.0(1.1)	1(0,4)	1.5(1.2)	1(0,4)	0.35(0.12–0.58)	39.1
Waste of time	0.1(0.4)	0(0,2)	0.1(0.4)	0(0,2)	0.38(-0.18–0.93)	95.3
Practical problems	0.5(1.0)	0(0,4)	0.7(1.3)	0(0,4)	0.20(-0.04–0.45)	76.6
Total**	8.2(4.4)	8(0,22)	10.2(5.1)	9(0,26)	--	--
Cronbach alpha	0.49		0.55		--	--

Significant difference between patient- and caregiver-ratings is labeled with *, *p < 0.01; **p < 0.001. p < 0.05 is considered statistically significant.

When we tested for agreement (using weighted Kappa) between patients and caregivers, we found agreement to be substantial for pain, moderate for four items (other symptoms, feeling anxious, family anxious and share feeling), fair for three items (life worthwhile, felt good and time wasted), and slight for two items (information given and practical problems). However, agreement for three of the items: information, time wasted and practical problems, should be interpreted cautiously, since the high level of percentage agreement for these items (71.9%, 95.3% and 76.6%) signaled that the weighted Kappa statistics may be affected by prevalence bias and thus underestimate the true agreement.

There was no significant correlation between caregiver burden and positivity (Rho = -0.16, p = 0.21), nor between total POS score and caregiver burden (Rho = 0.10, p = 0.45) or positivity (Rho = -0.08, P = 0.52). The disagreement between caregivers and patients over three POS items was significantly associated with caregiver burden (Table 3). Disagreement on three POS items were more likely to occur among caregivers with higher burden: "feeling anxious" (OR= 4.50; 95%CI: 1.30 to 15.59; P = 0.018), "life worthwhile"(OR = 12.43; 95%CI: 2.85 to 54.27; P = 0.001) and "felt good"(OR = 7.73; 95%CI: 2.02 to 29.60; P = 0.003). Caregivers with more positivity had higher agreement with the patients on "felt good" (OR = 0.27; 95% CI: 0.08 to 0.86; P = 0.027) than those with less positivity but were more likely to disagree (OR = 4.22; 95% CI: 1.08 to 16.55; P = 0.039) with patients for the POS item "practical problems". The two sensitivity analysis produced similar results, both varying cutoff for agreement and for burden and positivity, although some results were non-significant because of the smaller numbers. Unadjusted odds ratios also identified these associations, but they tended to underestimate the effects of caregiver burden and positivity on POS agreement.

**Table 3:** Adjusted odds ratios (95%CI) and unadjusted odds ratios (95%CI) of caregiver rating not equal to patient on the palliative outcomes of advanced cancer patients, caregivers with higher compared to lower scores of ZBI/positivity, results were derived from logistic regression models and adjusted or not adjusted for primary cancer site, caregiver age, caregiver sex, relationship to patient, patient living status and caregiver employment status (N = 64).

**Question**	Burden (higher vs lower)		Positivity (higher vs lower)	
	Adjusted-OR (95%CI)	Unadjusted-OR (95%)	Adjusted-OR* (95%CI)	Unadjusted-OR (95%CI)

Pain	0.53(0.14–2.02)	0.88(0.33 to 2.34)	1.80(0.52–6.23)	1.63 (0.61 to 4.40)
Other symptoms	0.53(0.16–1.78)	0.59(0.22 to 1.61)	2.38(0.71–8.02)	1.43(0.53 to 3.84)
Feeling anxious	4.50(1.30–15.59)*	3.18 (1.15 to 8.84)*	2.22(0.72–6.90)	1.29(0.48 to 3.44)
Family anxious	1.63(0.47–5.62)	2.04 (0.72 to 5.73)	1.14(0.34–3.86)	0.82(0.30 to 2.26)
Information given	1.99(0.58–6.91)	2.04 (0.67 to 6.21)	0.40(0.11–1.45)	0.46(0.15 to 1.43)
Share feeling	1.46(0.41–5.25)	1.00 (0.37 to 2.66)	2.50(0.65–9.53)	1.45(0.54 to 3.88)
Life worthwhile	12.43(2.85–54.27)**	6.61 (2.21 to 19.76)**	0.95(0.32–2.82)	0.89(0.33 to 2.37)
Felt good as a person	7.73(2.02–29.60)**	5.65(1.83 to 17.46)**	0.27(0.08–0.86)*	0.32(0.11 to 0.90)*
Practical problems	0.46(0.12–1.79)	0.64(0.20 to 2.07)	4.22(1.08–16.55)*	2.90(0.86 to 9.78)

Note: An odds ratio of greater than 1 indicates that disagreement was more likely when caregivers had higher burden or more positivity (median score as the cutoff). The modeled probability was the caregiver disagrees with patient's rating on palliative outcomes.

*p < 0.05, **p < 0.01; p < 0.05 is considered statistically significant.

Multiple logistic regression analyses also indicated that agreement on "share feeling" was lower where caregiver was older (OR = 1.16, 95% CI: 1.04 to 1.29, P = 0.007 in caregiver burden model and OR = 1.17, 95%CI: 1.05 to 1.30, P = 0.006 in positivity model for every one year increase respectively); female gender was associated with increased risk of disagreement for "pain" (OR = 1.97, 95%CI: 0.12 to 3.81, P = 0.036 in caregiver burden model and OR = 1.77, 95%CI: 0.04 to 3.50, P = 0.046 in positivity model) and "family anxious"(OR = 2.81, 95%CI: 0.74 to 4.88, P = 0.008 in caregiver burden model and OR = 2.95, 95%CI: 0.88 to 5.02, P = 0.005 in positivity model). The other factors had no significant independent impact on agreement.

## Discussion

This study examined the validity of caregiver responses when compared to advanced cancer patients self ratings. It explored whether the caregiver burden and positivity are associated with agreement between the caregiver and patient ratings. Three key findings emerged. First, overall caregivers in our study showed substantial agreement with patients for pain, and moderate to fair agreement for seven out of nine other items of our scale, POS. Our levels of percentage agreement were similar to those between clinician and cancer patient self-ratings when assessing symptoms[31], where the levels of disagreement did not affect treatment decisions. Therefore our results suggest that contemporary caregiver assessments are reasonably valid and reliable compared to patients self assessments, at least using the scale in this study, POS. POS could be completed by caregivers to give an assessment of patient concerns in clinical practice and in research, if patients are likely to become unable to make assessments. Our findings differ from other research, which has suggested caregivers rate pain and symptoms more severely than patients [32-34]. It may be that at this stage of illness caregivers agree more with patients self ratings, or that the higher validity we found may be a feature of POS, which uses more detailed definitions of severity and effect on the person, than the terms mild, moderate, severe, used in many scales.

Second, agreement was lowest for the more personal and psychological items such as: whether 'life was worthwhile', whether 'feelings could be shared' and whether the patient 'felt good about themselves', where caregivers recorded more problems than did the patients. It is interesting to note that caregivers were less likely to say patient felt their life was worthwhile than did patient's self ratings. When clinicians are discussing end of life treatment options with caregivers[13], they should be aware that caregivers may rate the life of the patient as less worthwhile than the patient themselves. Our results raise concerns about the use of caregivers as proxies when there are problems with a patient's mental capacity, and also for those who are promoting assisted suicide by caregivers. Third, for these aspects (and also for assessments of patient anxiety), caregivers were more likely to disagree with the patient self-rating when caregiver burden was higher. Assessments of how 'anxious' the patient, "life worthwhile" and "feeling good about self" were affected by total burden. Therefore, caregivers in much burdened circumstances may be prone to assess certain patient situations unreliably. Our findings support the work of Sands et al. [35] which found that higher caregiver burden was associated with discrepancies in ratings of quality of life; our data showed a similar pattern among cancer patients and their caregivers. However, our data suggest that it is the psychological components of quality of life, rather than symptoms, that are least reliable and most influenced by burden. Conversely, positivity was associated with improved agreement on whether the patient's life was worthwhile. However, surprisingly less burdened caregivers were less likely to agree about patients' practical needs. This finding could make sense clinically, in that less burdened caregivers may be less in touch with patients and less aware of their practical needs. These results would need to be confirmed in future studies.

Finally, the total burden scores were not very high in this sample. When developing the measure Zarit defined less than 21 as low burden, higher than our mean (median) score of 18.5 (17)[19,20]. This is a surprising finding, as this was a sample of terminally patients, most of whom died within the next few weeks and months. All of the patients and caregivers in our study were receiving support from specialist palliative care services, including multi-professional home care teams. Systematic reviews have suggested that these teams relieve patient symptoms and support caregivers[36]. The finding of relatively low caregiver burden in our sample supports these studies. However it may also be that the Zarit measure of burden, which was developed initially to study the caregivers of people with dementia[19], misses some important aspects of burden in cancer, especially in palliative care settings. We plan, therefore, to undertake further work to understand caregiver burden in cancer, the best way to measure it, what are the best services and methods of support to help caregivers. These results suggest that if clinicians or researchers do rely on caregiver assessments as a proxy for patient concerns they should also measure caregiver burden and may need to adjust for differences and disagreement.

Our study has some limitations, the most important of which is the number of confounding variables that we could adjust for. We were limited here by the sample size (although our study was large compared to many which include terminally ill patients and caregivers and powered to detect an OR about 3.0) and the range of variables collected. Ideally we would have liked to adjust for the duration of care and broader sociodemographic variables such as educational level and culture[10]. The quality of the relationship between patients and caregivers may also have been very important. Nevertheless we were able to adjust for basic variables including cancer site, gender, age, relationship to patient and whether the patient lived alone or not. Interestingly, gender appeared to have some effect, with women caregivers showing higher disagreement with patient self-ratings for the items 'pain' and 'family anxiety'. However, there were many more women than men caregivers in our sample, so this result should be interpreted with caution. For older patients there was also more disagreement on whether patients were able to share feelings. It may be that older patients are less likely to discuss how they are feeling and so making it difficult for their caregivers to assess this aspect of care[37]. A further problem with the study was that we had to dichotomize caregiver burden into high and low. Nevertheless, our sensitivity analysis, using a different cut off point of high/low burden supported these results. We should bear in mind that the nature of the data (ordinal, non-parametric) and the sample size would not allow us to divide the data into quartiles or quintiles. It may be that our analysis diluted the effect of burden, and the true effect is larger. However, our data suggests hypotheses that can be tested in larger studies. Due to the data limitations, we could not examine in further detail how the perceived burden directionally affects the agreement, but our results raise alarm for items such as "life worthwhile" and "feeling good", for which disagreement is more likely to occur when caregivers have relatively high burden or less positivity.

## Conclusion

Caregiver burden and positivity influence caregivers' assessments. We may need to adjust for differences and disagreement when using caregiver assessment as a proxy for patients' concerns. Caregivers' assessments of psychological aspects, especially whether patient's consider their life is worthwhile, may be least valid.

## Competing interests

The authors declare that they have no competing interests.

## Authors' contributions

Professor IH is the principal investigator and oversaw the whole project, IH is responsible for the completeness and accuracy of the data. Data analyses were co-designed by listed authors and performed by WG, The manuscript was drafted by IH with inputs from WG Both authors approved the final version, IH is the guarantor of the whole study.

## Appendix: Palliative outcome scale

[Additional file 1]

## Supplementary Material

Additional file 1 Palliative Outcome Scale. The file provided the original questionnaire used in this manuscript for assessing palliative outcomes. Click here for file

## References

[B1] Solano JP, Gomes B, Higginson IJ (2006). A comparison of symptom prevalence in far advanced cancer, AIDS, heart disease, chronic obstructive pulmonary disease and renal disease. J Pain Symptom Manage.

[B2] Ingleton C, Faulkner A (1995). Quality assurance in palliative care: some of the problems. Eur J Cancer Care (Engl ).

[B3] Diehr P, Johnson LL (2005). Accounting for missing data in end-of-life research. J Palliat Med.

[B4] Mittal V, Rosen J, Govind R, Degenholtz H, Shingala S, Hulland S, Rhee Y, Kastango KB, Mulsant BH, Castle N, Rubin FH, Nace D (2007). Perception gap in quality-of-life ratings: an empirical investigation of nursing home residents and caregivers. Gerontologist.

[B5] Sprangers MAG, Aaronson NK (1992). The role of health care providers and significant others in evaluating the quality of life of patients with chronic disease: a review. J Clin Epidemiol.

[B6] Tang ST, McCorkle R (2002). Use of family proxies in quality of life research for cancer patients at the end of life: a literature review. Cancer Invest.

[B7] Torti FM, Gwyther LP, Reed SD, Friedman JY, Schulman KA (2004). A multinational review of recent trends and reports in dementia caregiver burden. Alzheimer Dis Assoc Disord.

[B8] Kissane DW (2004). The challenge of discrepancies in values among physicians, patients, and family members. Cancer.

[B9] Zarit SH, Robertson SM (2006). Positive dimensions of mental health. Aging Ment Health.

[B10] Pinquart M, Sorensen S (2003). Associations of stressors and uplifts of caregiving with caregiver burden and depressive mood: a meta-analysis. J Gerontol B Psychol Sci Soc Sci.

[B11] Schofield HL, Murphy B, Herrman HE, Bloch S, Singh B (1997). Family caregiving: measurement of emotional well-being and various aspects of the caregiving role. Psychol Med.

[B12] Reich JW, Zautra AJ, Davis M (2003). Dimensions of affect relationships: Models and their integrative implications.. Reviews of General Psychology.

[B13] Goodwin DM, Higginson IJ, Myers K, Douglas HR, Normand CE (2003). Effectiveness of palliative day care in improving pain, symptom control, and quality of life. J Pain Symptom Manage.

[B14] Higginson IJ, Donaldson N (2004). Relationship between three palliative care outcome scales. Health and Quality of Life Outcomes.

[B15] Kind P, Dolan P, Gudex C, Williams A (1998). Variations in population health status: results from a United Kingdom national questionnaire survey. BMJ.

[B16] Hearn J, Higginson IJ (1999). Development and validation of a core outcome measure for palliative care: the palliative care outcome scale. Qual Health Care.

[B17] Brandt HE, Deliens L, van der Steen JT, Ooms ME, Ribbe MW, van der Wal G (2005). The last days of life of nursing home patients with and without dementia assessed with the Palliative care Outcome Scale. Palliat Med.

[B18] Stevens AM, Gwilliam B, A'Hern R, Broadley K, Hardy J (2005). Experience in the use of the palliative care outcome scale. Support Care Cancer.

[B19] Zarit SH, Reever KE, Bach-Peterson J (1980). Relatives of the impaired elderly: correlates of feelings of burden. Gerontologist.

[B20] Zarit SH, Orr RD, Zarit JM (1985). The Hidden Victims of Alzheimer's Disease: Families Under Stress.

[B21] Lazarus RS, Folkman S (1984). Stress, Appraisal, and Coping.

[B22] Lawton MP, Kleban MH, Moss M, Rovine M, Glicksman A (1989). Measuring caregiving appraisal. J Gerontol.

[B23] Cicchetti DV, Allison T (1971). A New Procedure for Assessing Reliability of Scoring EEG Sleep Recordings. American Journal of EEG Technology.

[B24] Byrt T, Bishop J, Carlin JB (1993). Bias, prevalence and kappa. J Clin Epidemiol.

[B25] Landis JR, Koch GG (1977). The measurement of observer agreement for categorical data. Biometrics.

[B26] Greenland S (1989). Modeling and variable selection in epidemiologic analysis. Am J Public Health.

[B27] Coen RF, O'Boyle CA, Coakley D, Lawlor BA (2002). Individual quality of life factors distinguishing low-burden and high-burden caregivers of dementia patients. Dement Geriatr Cogn Disord.

[B28] Resnizky S, Bentur N (2006). Can family caregivers of terminally ill patients be a reliable source of information about the severity of patient symptoms?. Am J Hosp Palliat Care.

[B29] Albinsson L, Strang P (2003). Existential concerns of families of late-stage dementia patients: questions of freedom, choices, isolation, death, and meaning. J Palliat Med.

[B30] Hunt CK (2003). Concepts in caregiver research. J Nurs Scholarsh.

[B31] Basch E, Iasonos A, McDonough T, Barz A, Culkin A, Kris MG, Scher HI, Schrag D (2006). Patient versus clinician symptom reporting using the National Cancer Institute Common Terminology Criteria for Adverse Events: results of a questionnaire-based study. Lancet Oncol.

[B32] Higginson IJ, McCarthy M (1993). Validity of the support team assessment schedule: do staffs' ratings reflect those made by patients or their families?. Palliat Med.

[B33] McPherson CJ, Wilson KG, Lobchuk MM, Brajtman S (2008). Family caregivers' assessment of symptoms in patients with advanced cancer: concordance with patients and factors affecting accuracy. J Pain Symptom Manage.

[B34] Siminoff LA, Rose JH, Zhang A, Zyzanski SJ (2006). Measuring discord in treatment decision-making; progress toward development of a cancer communication and decision-making assessment tool. Psychooncology.

[B35] Sands LP, Ferreira P, Stewart AL, Brod M, Yaffe K (2004). What explains differences between dementia patients' and their caregivers' ratings of patients' quality of life?. Am J Geriatr Psychiatry.

[B36] Higginson IJ, Finlay IG, Goodwin DM, Hood K, Edwards AG, Cook A, Douglas HR, Normand CE (2003). Is there evidence that palliative care teams alter end-of-life experiences of patients and their caregivers?. J Pain Symptom Manage.

[B37] Jansen J, van WJ, van DS, Heeren T, Bensing J (2007). Patient education about treatment in cancer care: an overview of the literature on older patients' needs. Cancer Nurs.

