# Supplementary figures and images for: Caregiver assessment of patients with advanced cancer: concordance with patients, effect of burden and positivity
Source: Health Qual Life Outcomes. 2008 Jun 2;6:42. doi: 10.1186/1477-7525-6-42 (PMC2435233; doi:10.1186/1477-7525-6-42)

**Appendix 1 Palliative Outcome Scale**
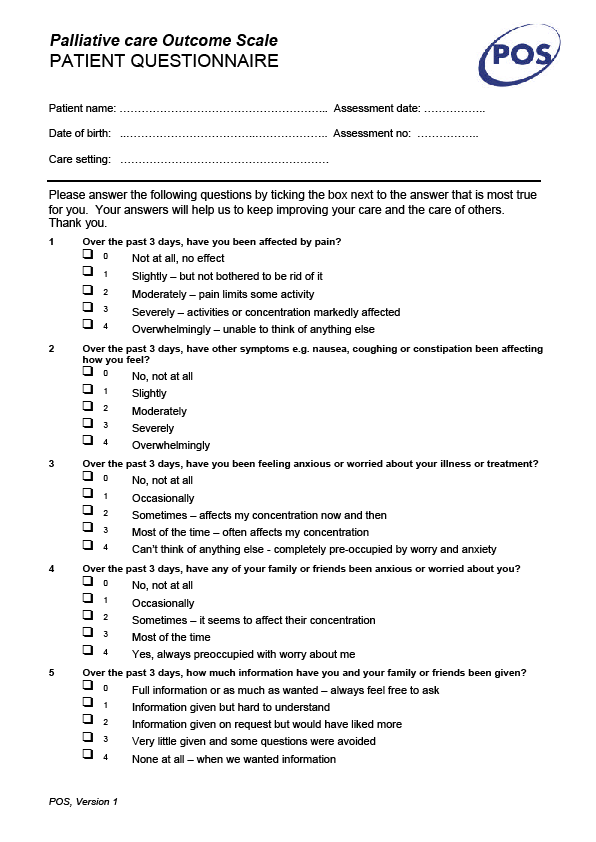

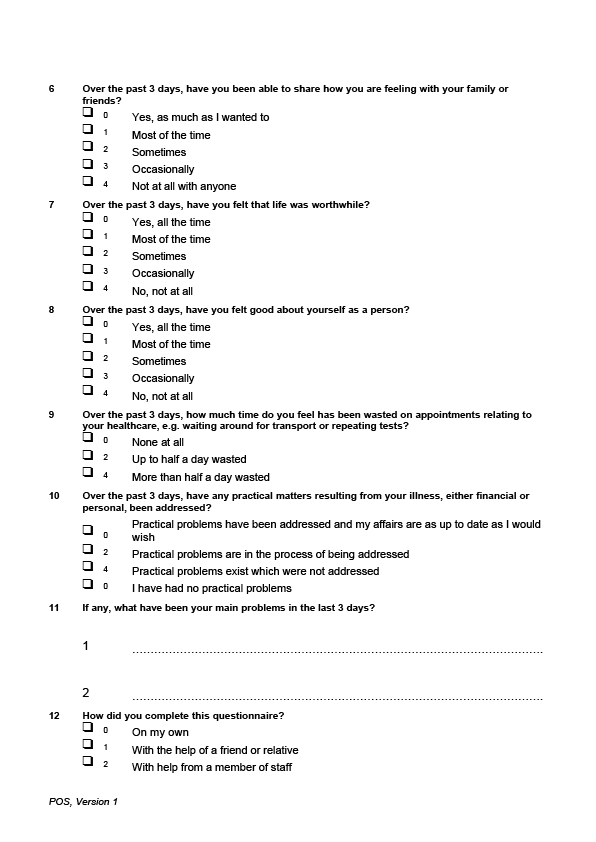

Supplement: Additional file 1 — Palliative Outcome Scale. The file provided the original questionnaire used in this manuscript for assessing palliative outcomes. [file 1477-7525-6-42-S1.doc]
